# The current state of prostate cancer in Antigua & Barbuda—2021

**DOI:** 10.3332/ecancer.2021.ed112

**Published:** 2021-08-17

**Authors:** Adrian R Rhudd

**Affiliations:** Department of Surgery, Mount St. John’s Medical Centre, Michael’s Mount, St. Johns, Antigua

**Keywords:** prostate cancer, Antigua, epidemiology, developing, current

## Abstract

Antigua and Barbuda is a twin-island nation within the Caribbean where the men are predominantly of African descent. The burden of prostate cancer is therefore expected to be similar to regional counterparts. There has been very little research done on prostate cancer in this nation. Few published peer-reviewed and non-peer reviewed documents exist to guide prostate cancer management and policy. A review of the available literature and the author’s experience managing prostate cancer in the country was used to provide a synopsis of prostate cancer management in the country.

All aspects of the prostate cancer care pathway exist in Antigua and Barbuda from public awareness and screening campaigns to external beam radiotherapy, abiraterone acetate and hospice care. There are still limitations to accessing some aspects of the care pathway such as prostate biopsies, radical prostatectomies and newer imaging modalities for staging. Data collection and analysis will help to provide objective and quantitative evidence of the nation’s prostate cancer management capabilities. Other developing nations will face many of the same challenges managing prostate and other cancers and this shared experience may be particularly useful for comparing and contrasting purposes. It also provides a documented milestone on which future plans can be made to improve care and shape national policy.

## Background

Prostate cancer (PCa) is the second commonest cancer diagnosed in men and is the fifth leading cause of cancer-related death in men globally [1]. Significant racial and geographic variations exist in the incidence, management and mortality of PCa [2]. In some populations, particularly those of African descent, it is the leading cause of cancer-related deaths [1].

The burden of prostate cancer is expected to persist and worsen to the extent that Rebbeck et al predict a doubling of prostate cancer related deaths between 2010 and 2030 [3]. In the 2018 Global Cancer Observatory (GLOBOCAN) update the Age Standardized incidence (ASIR) rate for PCa in the Caribbean was 64.2 per 100000 per year. This was sixth in the world out of the 21 World Health Organization (WHO) regions and the Age Standardized mortality (ASMR) rate was 25.4 per 100000 per year, which was fifth in the world out of the 21 WHO regions [1]. In 2021, the latest update to these figures shows an ASIR of 75.8 per 100000 per year, now the third highest in the world. ASMR for the Caribbean now stands at 27.9 per 100000 per year which is now the highest in the world. The current world average is 7.7 per 100,000 per year [4]. These figures however must be interpreted with caution as significant challenges exist with data collection and reporting in the developing nations [3].

Extensive research has been performed throughout the world to better understand this disease with a view to better diagnose, treat and lower the morbidity and mortality. Unfortunately, despite such an improved understanding, the improved care and management is not reflected in all populations.

Antigua and Barbuda (A&B) is a twin-island state in the Caribbean whose population is predominantly of African descent and as such the burden of prostate cancer in this population holds tremendous health and economic implications. The available published peer-reviewed and non-peer reviewed literature on prostate cancer epidemiology, diagnosis and management from this country is reviewed in this article. As a urologist with training and experience managing prostate cancer in larger nations such as Jamaica and the United Kingdom, the author also includes personal experience from managing prostate cancer in A&B for the past 18 months.

This article provides a concise synopsis of the prostate cancer care pathway in A&B to highlight capabilities of the healthcare system to manage this important disease. Other developing nations can compare and contrast this to their own PCa care pathways. This can help to stimulate dialogue, influence policy and ultimately improve care.

## Discussion

### Population of Antigua & Barbuda

A&B is a nation located in the north of the Caribbean. The United Nations Department of Economic and Social Affairs: Population Division puts the population in 2020 at just over 96,000, with about 45,000 being male [5]. The national census from 2013 showed that 91% of the population is “black” of African Descent [6]. The population pyramid demonstrated that in 2019 A&B had 16918 men between the ages of 40 and 74, the age recommended for PCa screening in a high-risk population [7]. This suggests that approximately 35% of all men in Antigua are eligible for PCa screening. The Pan American Health Organization (PAHO) Health in Americas, Country report on Antigua and Barbuda demonstrated that residents enjoy a good life expectancy of 75.2 years in men and 80.5 years in women and this was in 2015 [8].

### Local Prostate Cancer Epidemiology

There is no cancer registry or national cancer plan at the time of writing. Simon and colleagues published the latest peer-reviewed article on cancer epidemiology in A&B in 2014. For Prostate Cancer they found an ASIR of 69 per 100000 per year and also that 45% of all cancers diagnosed in men were from the prostate [9]. This figure was an increase from 22.8% in 1991, a figure described twenty three years earlier by the same author [10]. The Health Information Digest of 2015, published by the Ministry of Health reviewed the national mortality data from 2010-2012 and malignancies accounted for the leading cause of death in 2 of the three years. The number of deaths from all cancers, in men particularly, remained fairly constant, 58, 54 and 59 in 2010, 2011, and 2012 respectively [11]. In 2014 Daniel et al looked specifically at cancer mortality in the country and they found an ASMR for Prostate Cancer in Antigua to be 53.27-the period under review however was between 2001 and 2005 [12].

### Health Care system

Health care is delivered to patients in a 2-tiered approach, public health-care and private health-care. The government significantly subsidizes public health-care. The public health-care system delivers primary care via 25 health clinics within 3.2km of every community. Secondary and tertiary care is available at a single centre, the Mount St John’s Medical Centre (MSJMC) which has a capacity of 187 beds in Antigua. There is also an 8-bed facility in Barbuda [8].

The Medical Benefits Scheme (MBS) is a government run programme which allows eligible contributors to access subsidized health-care services. Most, if not all, employed and self-employed individuals in the country contribute between 2.5% and 7% of their wages to this scheme. This depends on age and wage. For employed individuals, this is split with up to 3.5% paid for by the employer. The scheme also allows beneficiaries to access refunds for services rendered at labs, imaging centres, and for surgical procedures as well as for some drugs [13]. Many of the prostate cancer management services fall under this scheme.

MSJMC is the main public referral centre for the country and as a result most patients requiring specialist care are seen here. This 185-bed hospital is the only tertiary institution where 3 urologists, 3 radiologists, 2 pathologists and, 2 oncologists can be accessed by Prostate Cancer patients. Most services here are subsidized by the MBS [14].

The Cancer Center Eastern Caribbean (TCCEC) is a private medical facility providing specialized oncology care to patients of A&B and the Eastern Caribbean. They offer private oncology consulting, CT scanning, Chemotherapy and External Beam Radiation Therapy (EBRT). Some of the services, particularly EBRT, is significantly subsidized by MBS [15]. This makes radiotherapy accessible and affordable to most men in the country. Monthly multidisciplinary tumor board meetings are conducted between MSJMC and TCCEC to discuss cancer cases, including PCa.

Two urologists are available for consultation in the private sector. Trans rectal ultrasound guided prostate biopsies are available there. Private histopathological analysis is available at one lab. Radical retropubic prostatectomies are also available privately. Two Private radiology centres exist offering ultrasound, Xray, CT and MRI services similar to MSJMC. Oncologists are available for consulting in the private health sector. Abiraterone acetate can be procured from abroad by private pharmaceutical distributors.

### Early detection and awareness

Currently there is no national screening programme for prostate cancer. Public campaigns on prostate cancer are run primarily in September and November to commemorate prostate cancer awareness month and men’s health month. Opportunistic screening with digital rectal examination (DRE) and prostatic specific antigen (PSA) is offered by private clinicians and at community clinics. Various government and private stakeholders host DRE and PSA screening events throughout the year. Interest in these screening events has steadily grown. The largest one is hosted by the Lions Club of Antigua and is held annually. The 13th installment of the event in 2019, had a record of almost 1000 men screened in one day.

### Diagnostics

Men with abnormal PSAs are referred in to public or private Urologists on a discretionary basis for further assessment, as no national protocol exists. The gold standard, trans-rectal ultrasound-guided biopsies are available only in the private sector. The availability of hardware and disposables to perform TRUS biopsies limits the availability in the public sector. Digital guided biopsies are infrequently performed at MSJMC when disposables are available. The majority of the prostate biopsies are therefore done in the private sector. Image guided biopsies and transperineal biopsies are not yet available on island. Histopathology assessment is provided by local pathologists at the MSJMC. Supplementary immunohistochemistry may be requested abroad from centres in Florida. Staging is performed using plain Xrays and CT scanning. As there is no nuclear medicine available on the island, bone scanning and PET scanning are not available. MRI machines are available but the local protocols and expertise to perform multiparametric MRI is not yet established.

### Treatment of localized disease

The primary modality of treatment for men with localized prostate cancer in A&B is external beam radiotherapy (EBRT) with androgen deprivation therapy (ADT). EBRT is administered at TCCEC. The pharmacological method of ADT available is with bicalutamide (Casodex®) and goserelin acetate (Zoladex®). These are also significantly subsidized by the MBS. Radical retro-pubic prostatectomy (RRP) is performed more frequently in the private sector. Most patients in the public sector present at an advanced disease state and age and are therefore not ideal candidates for RRP. Minimally invasive prostatectomy, brachytherapy and focal therapies are not available on island. Patients requesting these curative treatments have these done abroad.

### Treatment of advanced disease

ADT with Zoladex® after a two to four week course of Casodex® is the mainstay of treatment of advanced and metastatic disease. Docetaxel is available however it remains the primary choice for patients who progress to hormone refractory disease. It is not given “upfront” at diagnosis of metastatic disease as there is limited access to abiraterone acetate, enzalutamide, or apalutamide.

### Palliative care

The St. Johns Hospice was opened in Dec 2011 to offer palliative care services to patients and respite services to their relatives. At the time of opening it was the only institution in the Eastern Caribbean to offer such services. It is an 11-bed, non-profit charity institution [16]. Prostate cancer patients requiring end of life care have access to these facilities at a minimal cost.

## Conclusion

A&B is a twin-island nation within the Caribbean where the men are predominantly of African descent. The burden of prostate cancer is expected to be similar to regional counterparts. There are limited local data to drive policy and to guide a national approach to prostate cancer management. Men who are screened for prostate cancer and then go on to have a prostate biopsy have access to curative treatments mostly by way of EBRT+ ADT. This is heavily subsidized by the government’s MBS. Zoladex® is readily accessible for men with advanced disease and hospice care is available for men with end stage disease. There is limited access to trans rectal ultrasound guided prostate biopsy, radical retropubic prostatectomy in the public sector. More advanced imaging modalities to enhance staging are lacking. Access to a wider array of hormonal and second line agents to manage hormone refractory disease is limited. Understanding the current state of PCa management is important for making policies and plans to improve the care in the future.

## Conflicts of interest and funding

There are no conflicts of interest financial or otherwise to declare.
